# Effects of Progressive Inspiratory Muscle Training on Lung Volume and Respiratory Strength in Patients With Pre‐Dialysis Chronic Kidney Disease

**DOI:** 10.1002/pri.70320

**Published:** 2026-08-26

**Authors:** Mônica Soares de Oliveira, Tiago Moraes de Macêdo, Kaíque Ferreira Alves, Beatriz Luiza Marinho Cunha, Daiara Tathiana Xavier Nunes, Lilian Maria Melo da Silva, Patrícia Érika de Melo Marinho

**Affiliations:** ^1^ Post‐Graduation Program in Physical Therapy, Health Sciences Center Universidade Federal de Pernambuco Recife Pernambuco Brazil

**Keywords:** breathing exercises, chronic renal failure, lung volumes, plethysmography

## Abstract

**Introduction:**

Chronic kidney disease can present changes in thoracic cavity volume and respiratory muscle weakness, even in the pre‐dialysis stage. However, there is little evidence on the effects of inspiratory muscle training in these patients.

**Methods:**

This was a randomized clinical trial which comprised patients with Chronic Kidney Disease in stages 3, 4, and 5 undergoing conservative treatment, allocated experimental group (EG) with progressive loading (up to 50% of maximal inspiratory pressure [MIP]) and control group (CG) with a fixed load (5 cmH_2_O) and no load progression performed daily for 8 weeks. The outcomes assessed were thoracic cavity volumes, as measured by optoelectronic plethysmography, and inspiratory and expiratory respiratory muscle strength.

**Results:**

A total of 30 patients completed the study. All volumes significantly increased at the end of the protocol with a large effect size, but only the pulmonary thoracic cavity volume showed a significant interaction (*F* = 3.698; *p* = 0.042). There was an increase in inspiratory muscle strength in the EG (73.88–90.35 cmH_2_O; *p* < 0.001) and in the CG (70.85–86.92 cmH_2_O; *p* = 0.001), as well as in expiratory muscle strength in the EG (97.71–108.53 cmH_2_O; *p* = 0.001) and in the CG (80.69–94.77 cmH_2_O; *p* = 0.004).

**Conclusion:**

Daily IMT increased thoracoabdominal volumes, particularly pulmonary rib cage volume in the progressive‐load group, as well as respiratory muscle strength in patients with pre‐dialysis CKD. However, progressive‐load IMT was not superior to minimal‐load training.

## 1 Introduction

Chronic Kidney Disease (CKD) in the pre‐dialysis stage may be associated with repercussions in the respiratory system, leading to clinical and functional implications, and a worse prognosis in these patients (Gembillo et al. 2023; Mani et al. 2023). Therefore, alterations in pulmonary mechanics and respiratory muscle function have been described as systemic manifestations, reflecting in dyspnea upon exertion, exercise intolerance, fatigue, and increased susceptibility to respiratory complications (Katayifçi et al. 2024; Young et al. 2025).

In patients with CKD, fluid retention and volume overload contribute to the accumulation of fluid within the pulmonary interstitium, resulting in reduced lung compliance, increased work of breathing, and impaired gas exchange (Gembillo et al. 2023; Mayne et al. 2024). In addition, chronic uremia promotes structural and metabolic alterations in the respiratory muscles, compromising their contractile function and ultimately leading to respiratory muscle weakness (Heitman et al. 2024; Wang et al. 2022).

Pulmonary involvement manifests as changes in lung volumes and capacities, characterized by a reduction in vital capacity and total lung capacity, associated with decreased lung compliance and increased thoracic cavity stiffness (Mukai et al. 2018; Rocha et al. 2024). Uremic sarcopenia is also observed in parallel, affecting the respiratory muscles and leading to a reduction in inspiratory and expiratory muscle strength (Lin and Hsu 2021).

The presence of respiratory muscle weakness and reduced lung volumes were observed in a study involving hemodialysis patients who underwent an inspiratory muscle training (IMT) protocol (Medeiros et al. 2018). However, no studies to date have been found which have investigated the presence of changes in lung volumes, capacities and respiratory muscle weakness in CKD patients in the pre‐dialysis phase who underwent an IMT protocol.

Optoelectronic plethysmography (OEP) is a non‐invasive three‐dimensional imaging technique that provides a comprehensive assessment of lung volume changes, breathing patterns, and compartmental chest wall volume distribution. By enabling detailed evaluation of respiratory mechanics, OEP can detect abnormalities that may be present in patients with pre‐dialysis CKD. Thus, considering the repercussions of CKD on the respiratory system, the present study aimed to evaluate the effects of daily IMT on the variation in thoracic cavity volumes and respiratory muscle strength in CKD patients in the pre‐dialysis stage.

## 2 Method

This was a randomized clinical trial which followed the recommendations of the Consolidated Standards of Reporting Trials (CONSORT), approved by the Ethics and Research Committee of the Hospital das Clínicas of the Federal University of Pernambuco (Opinion No. 7,252,604) and registered in REBEC (U1111‐1320–2831). The norms of Resolution 466/12 of the National Health Council were respected, and all patients agreed to participate in the study by signing the informed consent form.

The study was conducted in the Cardiopulmonary Physiotherapy Laboratory from January 2025 to December 2025. Patients in the pre‐dialysis stage who were being followed up at the Nephrology Outpatient Clinic of the Hospital das Clínicas and the Barão de Lucena were recruited.

### 2.1 Sample Calculation

The sample size calculation was based on the results of the study by Katayifçi et al. (2024), which used maximum inspiratory pressure (MIP) as the main variable. The sample size calculation considered a power of 80%, an α of 5%, and an effect size of 0.21. A total of 15 patients were selected for each group (experimental and control). Considering possible losses during the study, 20% was added to this number, resulting in a sample of 18 patients for each group. The calculation was performed using the G*Power 3.1.9.2 statistical program (Faul et al. 2009).

### 2.2 Eligibility Criteria

Patients with a diagnosis of chronic kidney disease (CKD) undergoing pre‐dialysis treatment (stages 3, 4, and 5 non‐dialysis), aged 38–75 years, of both sexes, with the cognitive capacity to perform the assessment procedures and clinically and hemodynamically stable, were included. Individuals with respiratory diseases which altered pulmonary mechanics (asthma, COPD, bronchiectasis, history of tuberculosis), active infectious lung disease, or decompensated heart disease (acute or subacute worsening of signs and symptoms of heart failure) were excluded.

### 2.3 Allocation, Masking, and Randomization

Randomization was performed using randomization.com software by an independent researcher not involved in the study's assessment or intervention. A 1:1 allocation ratio was adopted between groups, with randomization in blocks. The generated sequence was kept confidential, and the allocation numbers were placed in opaque, black, sequentially numbered, and sealed envelopes, ensuring concealment of the allocation.

The envelopes were subsequently delivered to those responsible for training, who distributed the patients randomly assigned to either an experimental group (EG), which underwent inspiratory muscle training with progressive loading (up to 50% of maximal inspiratory pressure [MIP]), or a control group (CG), which performed inspiratory muscle training with a fixed load of 5 cmH_2_O without load progression. To minimize measurement bias, the outcome assessor remained blinded to group allocation throughout the study. Blinding of the physiotherapists responsible for administering the intervention was not feasible because they were necessarily aware of participants' treatment allocations.

Data were analyzed using a per‐protocol approach, including only participants who completed all study procedures. Participants who did not complete the intervention were considered lost to follow‐up and were excluded from the final analysis. No protocol deviations related to intervention delivery or load progression were identified among participants who completed the study.

### 2.4 Evaluation

Data regarding sex, height, weight, and body mass index (BMI) were collected, along with CKD history (time since diagnosis in months), disease stage (3, 4, and 5 non‐dialysis), etiology, presence of comorbidities, lifestyle habits (smoking and alcohol consumption), medication use, and laboratory tests.

### 2.5 Respiratory Muscle Strength

Respiratory muscle strength was assessed using a digital manometer (MVD 300, Globalmed, Porto Alegre, Brazil), with maximum inspiratory pressure (MIP) and maximum expiratory pressure (MEP) being measured before and after the IMT protocol, following the standards of the American Thoracic Society (ATS) and the European Respiratory Society (ERS) (American Thoracic Society/European Respiratory Society 2002).

At least three acceptable maneuvers were performed for each measurement in which they could not differ by more than 10%, and the highest value obtained was considered for analysis. The reliability of the test was considered adequate when at least 5 attempts were performed. The equations proposed by Neder et al. (1999) were used to calculate the predicted values for inspiratory (MIP) and expiratory (MEP) muscle strength. In turn, the following cut‐off points proposed by Caruso et al. (2015) were considered to classify respiratory muscle weakness: MIP < 60 cmH_2_O for women and < 80 cmH_2_O for men; MEP: < 120 cmH_2_O for women and < 150 cmH_2_O for men (Caruso et al. 2015).

### 2.6 Lung Volumes and Compartmental Distribution

Optoelectronic plethysmography (OEP) (BTS Bioengineering, Milan, Italy) is a valid and reliable method for assessing chest wall volumes (Cala et al. 1996). Its validity has been established through comparisons with spirometry, demonstrating excellent agreement for the measurement of lung volumes both at rest and during exercise (Cala et al. 1996). Reliability studies have also demonstrated high intra‐ and inter‐rater reproducibility for the main chest wall volume variables, with intraclass correlation coefficients exceeding 0.75 for most measurements (Cala et al. 1996; Vieira et al. 2013).

This was performed with patients seated on a stretcher, spine erect, hips and knees positioned at approximately 90°, feet supported, and their hands on rollers positioned beside their body to minimize thoracic cavity movement. Next, 89 infrared light‐reflective markers were fixed to the patient's skin, arranged in seven horizontal and five vertical lines in the thoracic‐abdominal region (Cala et al. 1996). Image acquisition was performed using six cameras positioned approximately 4 m anterior and posterior to the patient to ensure adequate marker reconstruction. The cameras captured the reflection of the markers, and the images of their displacement were transmitted to the computer using the OEP‐capture software, adopting a frequency of 60 Hz.

A parallel processor was used to recognize the patterns in real time, allowing for three‐dimensional (3D) reconstruction of the rib cage (Aliverti et al. 2000). The points formed a special geometric model developed to describe the surface of the entire thoracic wall and its different compartments: pulmonary rib cage, abdominal rib cage, and abdomen (Aliverti et al. 2001). The volumetric data of the area were calculated later using the SMART tracker software and analyzed in the Diamov software.

Patients initially breathed spontaneously for about 3 minutes. Then, the POWER‐breathe was used with a minimum inspiratory load (5 cmH2O), and patients were instructed to breathe for 1 minute. The equipment load was subsequently adjusted to 50% of the previously measured MIP for 1 minute. Assessments were performed before and after the intervention, enabling evaluation of the three‐compartment distribution of thoracic cavity volume both during resting breathing and resisted breathing.

The variables analyzed were: pulmonary thoracic cavity volume (*V*_PTC_), abdominal thoracic cavity volume (*V*_ATC_), and abdominal volume (*V*_AB_), expressed in liters. Total volume (*V*_T_) was expressed as the sum of the three compartments.

### 2.7 Inspiratory Muscle Training

The inspiratory muscle training protocol was performed daily for 30 minutes with POWERbreathe Classic Light (POWER breathe), over 8 weeks, performed at home. Training was performed once daily. Each session comprised 15 sets of 10 inspiratory breaths against the resistance provided by the device, with a 1‐min rest interval between sets.

Patients were instructed to perform the exercise in a seated position, using a nose clip and the mouthpiece of the device attached to the mouth (Pellizzaro et al. 2013). The sessions were recorded by the patients in a diary provided on the first day of training, which was checked weekly for adherence to the training.

The patients who were allocated to the EG started training with a load of 30% of their MIP, which was readjusted weekly with additional increments of 10% from the new MIP value obtained in the reassessment until reaching 50%. Upon reaching 50% of their MIP, the weekly reassessments maintained this value for recent MIP measurements until the end of the training. The CG maintained the minimum load of 5 cmH_2_O imposed by the equipment throughout the training period; however, they attended the sector weekly for reassessment of the MIP, without any change in load.

### 2.8 Statistical Analysis

Descriptive analysis was performed using mean and confidence interval measures for continuous quantitative variables and frequency and percentage for qualitative variables. Normality distribution was verified using the Shapiro‐Wilk test. Association measures were performed using Pearson's Chi‐squared test, and comparisons between the EG and CG were made using the *t*‐test for independent samples and the Mann‐Whitney test. The paired *t*‐test or Wilcoxon test was used to compare variables before and after the intervention. A per‐protocol analysis was performed, including only participants who completed all study procedures and had complete data for all prespecified assessments (Moher et al. 2010).

The analysis of variables related to total volume (*V*_T_), the rib cage pulmonary and abdominal compartment volumes (*V*_RCP_ and *V*_RCA_), and abdominal volume (*V*_AB_) was performed using repeated measures analysis of variance (ANOVA). The statistical model was designed considering two intra‐subject factors: the evaluation condition, structured in three levels (“No IMT”, “IMT/load 5cmH_2_O″ and “IMT/load 50%”), and the evaluation time, structured in two moments (initial—*T*_0_ and final—*T*_1_). These were crossed with an inter‐subject factor referring to the sample allocation group composed of two levels (“Experimental group” and “Control group”).

The assumption of sphericity of the covariance matrix was tested using Mauchly's Test. When this principle was violated (*p* < 0.05), the analysis was corrected using the Greenhouse‐Geisser estimate. Tukey's correction was used for multiple comparisons (post‐hoc) resulting from significant effects or interactions.

The effect size was reported using the partial squared Eta ($\eta_{p}^{2}$) to assess the magnitude of the difference and the strength of the relationships detected, being classified as follows: $\eta_{p}^{2}$ < 0.01 (small), $\eta_{p}^{2}$ between 0.02 and 0.06 (medium), and $\eta_{p}^{2}$ > 0.14 (large) (Cohen 1988).

Complete data processing was performed using the Jamovi statistical software (version 2.7), which operates based on the R programming language (version 4.5, R). All analyses were conducted assuming a significance level of 5% (*p* < 0.05).

## 3 Results

A total of 36 from the 174 patients eligible for the study were evaluated; however, six of them did not complete the study, totaling 30 patients in the sample. During follow‐up, one participant in the experimental group was lost due to a respiratory infection, and one participant in the control group was lost because of surgery. An additional four participants in the control group were lost to follow‐up because of the absence of a companion (*n* = 1) and lack of transportation (*n* = 3). All participants who completed the study adhered to the prescribed training protocol. No participant demonstrated intolerance to load progression that required modification of the intervention protocol during the study period. All training loads were adjusted based on the weekly reassessment of maximal inspiratory pressure (MIP). The training program was well accepted, and no adverse effects were reported.

**FIGURE 1:**
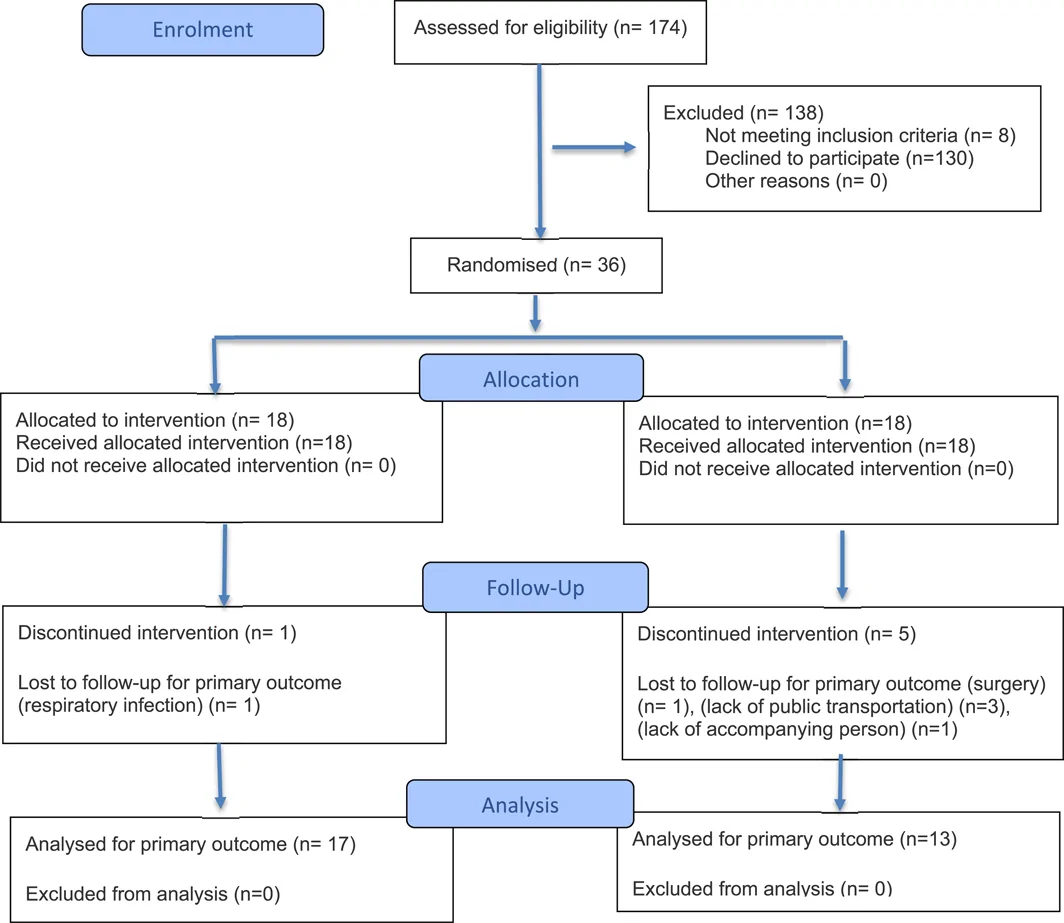
Flow diagram of the study.

A Figure 1 represents the patient flowchart in the study. Anthropometric, clinical, pulmonary function, and muscle strength data are shown in Table 1.

**TABLE 1:** Sociodemographic and clinical data of patients with non‐dialysis‐stage chronic kidney disease in the experimental and control groups.

Variables	Experimental group (*n* = 17) *n* (%)/mean (95% CI)	Control group (*n* = 13) *n* (%)/mean (95% CI)
Female sex	9 (52.9)	9 (69.2)
Age (years)	65.71 (60.03–71.38)	63 (56.21–71.38)
BMI (kg/m^2^)	28.87 (26.3–31.43)	26.43 (24.14–28.73)
Time since diagnosis (years)	3.88 (1.77–5.98)	3.02 (1.54–4.49)
Comorbidities		
SAH	17 (100)	11 (84.6)
DM	12 (70.6)	11 (84.6)
Medications		
Antihypertensive	17 (100)	11 (84.6)
Antianemic	2 (11.8)	2 (15.4)
Diuretic	3 (17.6)	3 (23.1)
Antidepressant	—	2 (15.4)
Hypoglycemic	11 (64.7)	6 (46.2)
Family history of CKD		
Yes	3 (17.6)	7 (53.8)
GFR	32.21 (24.44–37.97)	31.92 (26.07–37.77)
CKD stage		
3	8 (47.1)	6 (46.2)
4	6 (35.3)	7 (53.8)
5ND	3 (17.6)	—
Respiratory muscle strength		
MIP (cmH_2_O)	73.88 (58.82–88.95)	70.85 (51.16–90.53)
MEP (cmH_2_O)	97.71 (77.68–117.73)	80.69 (57.82–103.57)
% Respiratory muscle strength		
MIP (%)	77.49 (66.36–88.61)	70.23 (55.51–84.96)
MEP (%)	96.61 (85.01–108.14)	89.02 (73.41–104.62)
Spirometric parameters		
% FVC	63.53 (54.95–72.1)	61.46 (54.96–67‐96)
% FEV_1_	65.65 (56.14–75.16)	62.85 (54.1–71.6)
FEV_1_/FVC (L)	0.81 (0.74–0.88)	0.80 (0.75–0.84)

Abbreviations: BMI, Body Mass Index; CKD, Chronic Kidney Disease; DM, Diabetes Mellitus; FEV1, Forced Expiratory Volume in 1 Second; FVC, Forced Vital Capacity; GFR, Glomerular Filtration Rate; MEP, Maximum Expiratory Pressure; MIP, Maximum Inspiratory Pressure; ND, Non‐Dialysis; SAH, Systemic Arterial Hypertension.

The distribution of volumes by compartments (*V*_T_, *V*_RCP_, *V*_RCA_, and *V*_AB_) among the groups, the three conditions (No IMT, IMT/5 cmH_2_O, and IMT/50%), and time point (initial and final) are described and detailed in Table 2.

**TABLE 2:** Behavior of volumes between the experimental and control groups at the initial and final moments, according to the evaluation performed without IMT, with IMT/5 cmH_2_O and IMT/50%.

Variables	Groups	No IMT			IMT/5 cmH2O			IMT/50%		
		Evaluation	Re‐evaluation	*p* value	Evaluation	Re‐evaluation	*p* value	Evaluation	Re‐evaluation	*p* value
*V*_T_ (mL)	Experimental	0.461 ± 0.191	0.451 ± 0.162	0.780^b^	0.887 ± 0.704	0.947 ± 0.617	0.602^b^	0.716 ± 0.280	0.755 ± 0.394	0.603^b^
	Control	0.555 ± 0.165	0.617 ± 0.242	0.129^b^	0.978 ± 0.514	0.907 ± 0.468	0.443^b^	1.039 ± 0.479	1.136 ± 0.49	0.168^b^
	*p* value	0.165^a^	0.032^a^	—	0.698^a^	0.849^a^	—	0.028^a^	0.025^a^	—
*V*_RCP_ (mL)	Experimental	0.151 ± 0.084	0.131 ± 0.064	0.177^b^	0.241 ± 0.343	0.275 ± 0.321	0.353^b^	0.227 ± 0.254	0.237 ± 0.254	0.697^b^
	Control	0.219 ± 0.181	0.271 ± 0.186	0.041^b^	0.329 ± 0.206	0.283 ± 0.154	0.268^b^	0.360 ± 0.197	0.409 ± 0.244	0.180^b^
	*p* value	0.178^a^	0.021^a^	—	0.425^a^	0.936^a^	—	0.129^a^	0.072^a^	—
*V*_RCA_ (mL)	Experimental	0.081 ± 0.066	0.084 ± 0.058	0.847^b^	0.174 ± 0.152	0.188 ± 0.126	0.370^b^	0.170 ± 0.185	0.167 ± 0.185	0.841^b^
	Control	0.095 ± 0.039	0.098 ± 0.044	0.356^b^	0.170 ± 0.112	0.171 ± 0.104	0.943^b^	0.167 ± 0.185	0.228 ± 0.125	0.801^b^
	*p* value	0.515^a^	0.462^a^	—	0.948^a^	0.691^a^	—	0.276^a^	0.320^a^	—
*V*_AB_ (mL)	Experimental	0.237 ± 0.122	0.245 ± 0.138	0.678^b^	0.419 ± 0.349	0.488 ± 0.323	0.425^b^	0.425 ± 0.297	0.457 ± 0.331	0.495^b^
	Control	0.241 ± 0.104	0.248 ± 0.150	0.691^b^	0.477 ± 0.294	0.452 ± 0.331	0.676^b^	0.459 ± 0.290	0.498 ± 0.278	0.058^b^
	*p* value	0.936^a^	0.948^a^	—	0.634^a^	0.769^a^	—	0.753^a^	0.717^a^	—

*Note:* Data presented as mean ± standard deviation.

Abbreviations: IMT, inspiratory muscle training; *T*_0_, initial assessment; *T*_1_, final assessment; *V*_AB_, abdominal volume; *V*_RCA_, rib cage abdominal; *V*_RCP_, rib cage pulmonary volume; *V*_T_, total volume.

^a^ Student's *t*‐test for independent samples.

^b^ Student's *t*‐test for paired samples.

### 3.1 *V*_T_

A change in thoracic capacity volume was observed when treatment levels (no IMT, IMT/5 cmH_2_O, and IMT/50%) were compared (F with Greenhouse‐Geisser correction = 15.383; *p* < 0.001), with a large effect size ($\eta_{p}^{2}$ = 0.355). No differences were observed at the final time point between the initial and final time points (*F* = 0.726; *p* = 0.402), nor was a “treatment × time × group” interaction detected (*p* = 0.299, with Greenhouse‐Geisser correction). Table 2 presents the results of the analyses performed.

### 3.2 *V*_RCP_

A change was observed for the *V*_RCP_ between treatment levels (No IMT, IMT/5 cmH_2_O and IMT/50%), F with Greenhouse‐Geisser correction = 4.289; *p* = 0.029, with a large effect size ($\eta_{p}^{2}$ = 0.133), with no difference between the initial and final time points (*F* = 1.207; *p* = 0.281). A treatment × time × group interaction was observed (*F* with Greenhouse‐Geisser correction = 3.698; *p* = 0.042).

### 3.3 *V*_RCA_

It was observed for the *V*_RCA_ that the different treatment levels (No IMT, IMT/5 cmH_2_O and IMT/50%) produced distinct responses (F with Greenhouse‐Geisser correction = 11.624; *p* < 0.001) and a large effect size ($\eta_{p}^{2}$ = 0.293). No differences were observed between the initial and final time points (*F* = 0.199; *p* = 0.659), nor was the presence of a treatment × time × group interaction found (*F* = 0.112; *p* = 0.894).

### 3.4 *V*_AB_

For *V*_AB_, it was observed that the different treatment levels (No IMT, IMT/5 cmH_2_O and IMT/50%) produced distinct responses (*F* = 16.6421; *p* < 0.001) with a large effect size ($\eta_{p}^{2}$ = 0.373); however, no differences were observed between the initial and final moments (*F* = 0.7486; *p* = 0.394), nor was a treatment × moment × group interaction detected (*F* = 0.7896; *p* = 0.411, with Greenhouse‐Geisser correction).

Finally, an increase in MIP was observed in both the EG (73.88–90.35 cmH_2_O, *p* < 0.001) and the CG (70.85–86.92 cmH_2_O, *p* = 0.001) at the end of the study. Similarly, there was an increase in MEP in the EG (97.71–108.53 cmH_2_O, *p* = 0.001) and in the CG (80.69–94.77 cmH_2_O, *p* = 0.004). However, no differences were observed between the groups. No significant between‐group differences were observed for any outcome, except for pulmonary rib cage volume (*V*_RCP_).

## 4 Discussion

This study evaluated the behavior of thoracic cavity volumes and respiratory muscle strength in pre‐dialysis CKD patients after an 8‐week IMT protocol. Regardless of the training load, IMT was sufficient to promote volume changes in the thoracic‐abdominal compartments, particularly in the pulmonary rib cage compartment, accompanied by increased respiratory muscle strength in both groups. However, incremental inspiratory muscle training did not demonstrate superiority over the control group.

### 4.1 Thoracic Cavity Volumes

According to the results presented, all patients responded favorably to the change in volume between the thoracic cavity compartments at the end of the training, irrespective of the imposed training load. Although thoracic cavity distensibility depends on pulmonary compliance and the ability of inspiratory muscles to contract, the presence of chronic pulmonary edema, lung parenchymal calcifications, and interstitial fibrosis resulting from impaired mineral and bone metabolism in CKD should be considered, even at these stages of kidney disease (Medeiros et al. 2018; Patel et al. 2025; Siracusa et al. 2024).

The institution of an IMT program with lower loads may facilitate patient adherence to training programs for strengthening inspiratory muscles, especially for those who are intolerant to physical exertion (Al‐Otaibi et al. 2024).

Our results differ from those described by Medeiros et al. (2018), who found better performance for the group of patients with CKD on hemodialysis who trained with 50% load. It is expected that as kidney disease progresses, factors such as increased volume overload, pulmonary interstitial edema, and fibrotic remodeling of the lung parenchyma (Mayne et al. 2024; Kishi et al. 2024), combined with mineral metabolism disorders, may contribute to calcify the lung parenchyma, limiting thoracic expansion in these patients (Siracusa et al. 2024; Ketteler et al. 2025), resulting in less volumetric variation of the thorax (Patel et al. 2025; Gembillo et al. 2023), thereby justifying the higher training load among hemodialysis patients.

According to the results of our study, a significant variation in lung volumes was observed when patients were evaluated in spontaneous breathing with a load of 5 cmH_2_O and 50% of MIP. This result is expected when inspiratory resistance is applied, which not only promotes greater muscle recruitment but also a greater increase in lung volume proportional to the imposed load, a phenomenon observed in healthy individuals (Gama et al. 2013; Manifield et al. 2024). This behavior has also been observed in individuals with other chronic health conditions (Lage et al. 2018; Myrrha et al. 2013) who showed an increase in thoracic‐abdominal volume under resisted breathing.

### 4.2 Respiratory Muscle Strength

Our findings demonstrate that both the group trained with a fixed minimal inspiratory load and the group that underwent weekly load adjustments according to the principles of individualization and progressive overload exhibited significant improvements in maximal inspiratory pressure (MIP) and maximal expiratory pressure (MEP) following the IMT intervention, which was also observed in previous studies (Medeiros et al. 2018; Katayifçi et al. 2024).

According to our findings, progressive loading may not be the sole determinant of the muscular adaptations observed in this study. Minimal‐load training protocols, provided they incorporate an adequate training volume through repeated contractions (Ikezoe et al. 2020), may be sufficient to induce adaptations in the respiratory muscles. This may be particularly relevant for patients with CKD, in whom training with higher loads may be poorly tolerated or even contraindicated.

In patients with pre‐dialysis CKD in this study, the training stimulus applied may have been sufficient to improve respiratory muscle strength. However, inspiratory and/or expiratory muscle weakness was not reversed in all participants. This finding highlights the need to consider patient‐specific characteristics, such as CKD duration, comorbidities, disease stage, and other clinical factors, when prescribing exercise. Optimizing exercise prescription according to the FITT‐VP principles (frequency, intensity, time, type, volume, and progression) may be necessary to maximize training‐induced adaptations and achieve more robust clinical outcomes.

The greater participation of the rib cage muscles and significant activation of the abdominal muscles may be due to stretching of the expiratory muscles during deep inspiration performed with a load, thus favoring an increase in strength during expiration (Aliverti et al. 1997; Ratnovsky et al. 2008), thus justifying the increase in expiratory muscle strength in our study.

### 4.3 Lung Function

Patients with CKD enrolled in the present study exhibited predicted forced vital capacity (FVC) and forced expiratory volume in one second (FEV_1_) values below the normal reference range, indicating impaired pulmonary function during the pre‐dialysis stage, in agreement with previous studies (Rezende et al. 2022; Mukai et al. 2018). Although CKD is typically associated with a predominantly restrictive ventilatory pattern resulting from reduced lung compliance, increased chest wall stiffness, respiratory muscle dysfunction, and chronic systemic inflammation (Navaneethan et al. 2016; Mukai et al. 2018), obstructive ventilatory abnormalities were also observed, despite preservation of the FEV_1_/FVC ratio. Irrespective of the ventilatory pattern identified, these findings reinforce that pulmonary impairment is already evident in the early stages of CKD, before the initiation of kidney replacement therapy.

The smaller sample size than that proposed in this study may constitute a possible limitation for generalizing the results presented. A potential limitation of the present study is that adherence to the home‐based training program was monitored using training diaries and weekly follow‐up rather than objective monitoring methods. Additionally, the absence of functional outcome measures may have limited the evaluation of the clinical relevance and functional implications of the proposed training protocol. Future studies should include functional outcome measures to enhance the clinical applicability of IMT in patients with pre‐dialysis CKD, and the findings of the present study should therefore be interpreted with caution.

Another limitation of the present study was the differential attrition observed in the control group following randomization. Although this may have introduced attrition bias (Moher et al. 2010), participant withdrawal was primarily due to reasons unrelated to the intervention, including the need for surgery and difficulties traveling to the study site, as documented in the CONSORT flow diagram. Nevertheless, the resulting loss to follow‐up may have reduced the study's statistical power and affected the robustness of the findings. Accordingly, the results should be interpreted with appropriate caution.

Finally, IMT is currently used in clinical practice for other health conditions; however, this has not been observed in patients with CKD, especially in the pre‐dialysis stages. In this sense, the present study draws attention to the benefits derived from IMT, particularly in this sample of patients.

## 5 Final Considerations

Daily IMT increased thoracoabdominal volumes, particularly pulmonary rib cage volume in the progressive‐load group, as well as respiratory muscle strength in patients with pre‐dialysis CKD. However, progressive‐load IMT was not superior to minimal‐load training.

## 6 Implications of Physiotherapy Practice

Considering that patients with pre‐dialysis chronic kidney disease (CKD) exhibit functional impairments associated with respiratory muscle weakness, the implementation of an individualized inspiratory muscle training (IMT) program may improve respiratory muscle function before progression to end‐stage kidney disease. Furthermore, this intervention can be performed at home with weekly supervision, making it a feasible and well‐tolerated strategy for this patient population.

## Author Contributions

**Mônica Soares de Oliveira:** conceptualization, methodology, investigation, data curation, formal analysis, writing – original draft. **Tiago Moraes de Macêdo:** methodology, formal analysis, writing – review and editing. **Kaique Ferreira Alves:** investigation, data curation, validation. **Beatriz Luiza Marinho Cunha:** investigation, data curation. **Daiara Tathiana Xavier Nunes:** formal analysis, validation. **Lilian Maria Melo da Silva:** supervision, project administration. **Patrícia Érika de Melo Marinho:** conceptualization, supervision, project administration, writing – review and editing, and final approval of the manuscript.

## Funding

This work was supported by the PROPESQI Call No. 06/2024, Institutional Research Productivity Grant Program.

## Ethics Statement

This study was approved by the Ethics and Research Committee of the Hospital das Clínicas of the Federal University of Pernambuco (Opinion No. 7,252,604).

## Conflicts of Interest

The authors declare no conflicts of interest.

## Data Availability

The The data that support the findings of this study are available on request from the corresponding author. The data are not publicly available due to privacy or ethical restrictions.

## References

[pri70320-bib-0001] Aliverti, A. , S. J. Cala , R. Duranti , et al. 1997. “Human Respiratory Muscle Actions and Control During Exercise.” Journal of Applied Physiology 83, no. 4: 1256–1269. 10.1152/jappl.1997.83.4.1256. 9338435

[pri70320-bib-0002] Aliverti, A. , R. Dellacá , P. Pelosi , D. Chiumello , L. Gattinoni , and A. Pedotti . 2001. “Compartmental Analysis of Breathing in the Supine and Prone Positions by Optoelectronic Plethysmography.” Annals of Biomedical Engineering 29, no. 1: 60–70. 10.1114/1.1332084. 11219508

[pri70320-bib-0003] Aliverti, A. , R. Dellacá , P. Pelosi , D. Chiumello , A. Pedotti , and L. Gattinoni . 2000. “Optoelectronic Plethysmography in Intensive Care Patients.” American Journal of Respiratory and Critical Care Medicine 161, no. 5: 1546–1552. 10.1164/ajrccm.161.5.9903024. 10806152

[pri70320-bib-0004] Al‐Otaibi, H. M. , F. Sartor , and H. P. Kubis . 2024. “The Influence of Low Resistance Respiratory Muscle Training on Pulmonary Function and High Intensity Exercise Performance.” Journal of Exercise Science & Fitness 22, no. 3: 179–186. 10.1016/j.jesf.2024.02.007. 38495300 PMC10937314

[pri70320-bib-0005] American Thoracic Society/European Respiratory Society . 2002. “ATS/ERS Statement on Respiratory Muscle Testing.” American Journal of Respiratory and Critical Care Medicine 15, no. 4: 518–624. 10.1164/rccm.166.4.518. 12186831

[pri70320-bib-0006] Cala, S. J. , C. M. Kenyon , G. Ferrigno , et al. 1996. “Chest Wall and Lung Volume Estimation by Optical Reflectance Motion Analysis.” Journal of Applied Physiology 81, no. 6: 2680–2689. 10.1152/jappl.1996.81.6.2680. 9018522

[pri70320-bib-0007] Caruso, P. , A. L. P. Albuquerque , P. V. Santana , et al. 2015. “Diagnostic Methods to Assess Inspiratory and Expiratory Muscle Strength.” Jornal Brasileiro de Pneumologia 41, no. 2: 110–123. 10.1590/s1806-37132015000004474. 25972965 PMC4428848

[pri70320-bib-0008] Cohen, J. 1988. Statistical Power Analysis for the Behavioral Sciences. 2nd ed. Erlbaum.

[pri70320-bib-0009] Faul, F. , E. Erdfelder , A. Buchner , and A. G. Lang . 2009. “Statistical Power Analyses Using G*Power 3.1: Tests for Correlation and Regression Analyses.” Behavior Research Methods 41, no. 4: 1149–1160. 10.3758/brm.41.4.1149. 19897823

[pri70320-bib-0010] Gama, A. E. F. , L. A. Carvalho , and L. A. Feitosa . 2013. “Acute Effects of Incremental Inspiratory Loads on Compartmental Chest Wall Volume and Predominant Activity Frequency of Inspiratory Muscle.” Journal of Electromyography and Kinesiology 23, no. 6. 10.1016/j.jelekin.2013.07.014. 24035013

[pri70320-bib-0011] Gembillo, G. , S. Calimeri , V. Tranchida , et al. 2023. “Lung Dysfunction and Chronic Kidney Disease: A Complex Network of Multiple Interactions.” Journal of Personalized Medicine 13, no. 2: 1–23. 10.3390/jpm13020286. PMC9966880 36836520

[pri70320-bib-0012] Heitman, K. , M. S. Alexander , and C. Faul . 2024. “Skeletal Muscle Injury in Chronic Kidney Disease‐From Histologic Changes to Molecular Mechanisms and to Novel Therapies.” International Journal of Molecular Sciences 8, no. 10: 25. 10.3390/ijms25105117. PMC11121428 38791164

[pri70320-bib-0013] Ikezoe, T. , T. Kobayashi , M. Nakamura , and N. Ichihashi . 2020. “Effects of Low‐Load, Higher‐Repetition vs. High‐Load, Lower‐Repetition Resistance Training Not Performed to Failure on Muscle Strength, Mass, and Echo Intensity in Healthy Young Men: A Time‐Course Study.” Journal of Strength & Conditioning Research 34, no. 12: 3439–3445. 10.1519/jsc.0000000000002278. 29016473

[pri70320-bib-0014] Katayifçi, N. , I. Huzmeli , D. Iri , et al. 2024. “Effects of Different Inspiratory Muscle Training Protocols on Functional Exercise Capacity and Respiratory and Peripheral Muscle Strength in Patients With Chronic Kidney Disease: A Randomized Study.” BMC Nephrology 25, no. 18: 1–10. 10.1186/s12882-024-03610-1. 38811888 PMC11137907

[pri70320-bib-0015] Ketteler, M. , P. Evenepoel , R. Holden , et al. 2025. “Chronic Kidney Disease‐Mineral and Bone Disorder: Conclusions From a Kidney Disease: Improving Global Outcomes (KDIGO) Controversies Conference.” Kidney International 107, no. 3: 405–423. 10.1016/j.kint.2024.11.013. 39864017

[pri70320-bib-0016] Kishi, S. , H. Nagasu , K. Kidokoro , and N. Kashihara . 2024. “Oxidative Stress and the Role of Redox Signalling in Chronic Kidney Disease.” Nature Reviews Nephrology 20, no. 2: 101–119. 10.1038/s41581-023-00775-0. 37857763

[pri70320-bib-0017] Lage, S. M. , R. R. Britto , D. C. Brandão , D. A. G. Pereira , A. D. d. Andrade , and V. F. Parreira . 2018. “Can Diaphragmatic Breathing Modify Chest Wall Volumes During Inspiratory Loaded Breathing in Patients With Heart Failure?” Brazilian Journal of Physical Therapy 22, no. 6: 452–458. 10.1016/j.bjpt.2018.04.005. 29752160 PMC6235751

[pri70320-bib-0018] Lin, Y. L. , and B. G. Hsu . 2021. “Assessment of Uremic Sarcopenia in Dialysis Patients: An Update.” Tzu Chi Medical Journal 34, no. 2: 182–191. 10.4103/tcmj.tcmj_254_20. 35465288 PMC9020246

[pri70320-bib-0019] Mani, P. A. , R. Sundar , and S. Yadav . 2023. “Pulmonary Manifestations at Different Stages in Chronic Kidney Disease: An Observational Study.” Cureus 15, no. 19: 1–7. 10.7759/cureus.39235. PMC10277162 37337495

[pri70320-bib-0020] Manifield, J. , C. Alexiou , D. Megaritis , et al. 2024. “Effects of Inspiratory Muscle Training on Thoracoabdominal Volume Regulation in Older Adults: A Randomised Controlled Trial.” Respiratory Physiology & Neurobiology 326: 1–13. 10.1016/j.resp.2024.104278. 38735425

[pri70320-bib-0021] Mayne, K. J. , N. Staplin , D. F. Keane , et al. 2024. “Effects of Empagliflozin on Fluid Overload, Weight, and Blood Pressure in CKD.” Journal of the American Society of Nephrology 35, no. 2: 202–215. 10.1681/asn.0000000000000271. 38082486 PMC7615589

[pri70320-bib-0022] Medeiros, A. I. C. , H. K. B. Fuzari , C. Rattes , et al. 2018. “Inspiratory Muscle Training Improves Respiratory Muscle Strength, Functional Capacity and Quality of Life in Patients With Chronic Kidney Disease: A Systematic Review.” Journal of Physiotherapy 63, no. 1: 76–86. 10.1016/j.jphys.2017.02.016. 28433237

[pri70320-bib-0023] Moher, D. , S. Hopewell , K. F. Schulz , et al. 2010. “CONSORT 2010 Explanation and Elaboration: Updated Guidelines for Reporting Parallel Group Randomised Trials.” International Journal of Surgery 10, no. 1: 28–55. 10.1016/j.ijsu.2011.10.001. 22036893

[pri70320-bib-0024] Mukai, H. , P. Ming , B. Lindholm , et al. 2018. “Restrictive Lung Disorder Is Common in Patients With Kidney Failure and Associates With Protein‐Energy Wasting, Inflammation and Cardiovascular Disease.” PLoS One 13, no. 4: e0195585. 10.1371/journal.pone.0195585. 29702682 PMC5922538

[pri70320-bib-0025] Myrrha, M. A. C. , D. S. R. Vieira , K. S. Moraes , et al. 2013. “Chest Wall Volumes During Inspiratory Loaded Breathing in COPD Patients.” Respiratory Physiology & Neurobiology 188, no. 1. 10.1016/j.resp.2013.04.017. 23628707

[pri70320-bib-0026] Navaneethan, S. D. , S. Mandayam , S. Arrigain , M. Rahman , W. C. Winkelmayer , and J. D. Schold . 2016. “Obstructive and Restrictive Lung Function Measures and CKD: National Health and Nutrition Examination Survey (NHANES) 2007–2012.” American Journal of Kidney Diseases 68, no. 3: 414–421. 10.1053/j.ajkd.2016.03.415. 27130720 PMC5002238

[pri70320-bib-0027] Neder, J. A. , S. Andreoni , M. C. Lerario , and L. Nery . 1999. “Reference Values for Lung Function Tests. II. Maximal Respiratory Pressures and Voluntary Ventilation.” Brazilian Journal of Medical and Biological Research 32, no. 6: 719–727. 10.1590/s0100-879x1999000600007. 10412550

[pri70320-bib-0028] Patel, I. , J. Zhang , Y. Chai , et al. 2025. “Preserved Ratio Impaired Spirometry, Airflow Obstruction, and Their Trajectories in Relationship to Chronic Kidney Disease: A Prospective Cohort Study.” Scientific Reports 15, no. 1: 3439. 10.1038/s41598-025-86952-6. 39870785 PMC11772821

[pri70320-bib-0029] Pellizzaro, C. O. , F. S. Thomé , and F. V. Veronese . 2013. “Effect of Peripheral and Respiratory Muscle Training on the Functional Capacity of Hemodialysis Patients.” Renal Failure 35, no. 2: 189–197. 10.3109/0886022x.2012.745727. 23199095

[pri70320-bib-0030] Ratnovsky, A. , D. Elad , and P. Halpern . 2008. “Mechanics of Respiratory Muscles.” Respiratory Physiology & Neurobiology 163, no. 1–3: 1–3. 10.1016/j.resp.2008.04.019. 18583200

[pri70320-bib-0031] Rezende, P. S. , F. Andrade , C. Borba , and P. M. Rovedder . 2022. “Pulmonary Function, Muscle Strength, and Quality of Life Have Differed Between Chronic Kidney Disease Patients and Healthy Individuals.” Therapeutic Apheresis and Dialysis 26: 337–344. 10.1111/1744-9987.13714. 34328280

[pri70320-bib-0032] Rocha, L. G. , J. H. Policarpo , B. L. M. Cunha , J. R. Silva , and P. É. Marinho . 2024. “Muscle Strength and Pulmonary Function in Individuals With Chronic Kidney Disease: A Cross‐Sectional Study.” Manual Therapy, Posturology & Rehabilitation Journal 22. 10.17784/mtprehabjournal.2024.22.1321.

[pri70320-bib-0033] Siracusa, C. , N. Carabetta , M. B. Morano , et al. 2024. “Understanding Vascular Calcification in Chronic Kidney Disease: Pathogenesis and Therapeutic Implications.” International Journal of Molecular Sciences 25, no. 23: 13096. 10.3390/ijms252313096. 39684805 PMC11642360

[pri70320-bib-0034] Vieira, D. S. R. , M. Hoffman , D. A. G. Pereira , R. R. Britto , and V. F. Parreira . 2013. “Optoelectronic Plethysmography: Intra‐Rater and Inter‐Rater Reliability in Healthy Subjects.” Respiratory Physiology & Neurobiology 189, no. 3: 473–476. 10.1016/j.resp.2013.08.023. 24036178

[pri70320-bib-0035] Wang, B. , Z. J. Li , Y. L. Zhang , Y. Wen , Y. M. Gao , and B. C. Liu . 2022. “Hypoxia And Chronic Kidney Disease.” EBioMedicine 77: 1–11. 10.1016/J.EBIOM.2022.103942. PMC8921539 35290825

[pri70320-bib-0036] Young, H. M. L. , R. E. Billany , G. M. P. M. Brown , C. J. Lightfoot , D. S. March , and A. C. Smith . 2025. “Physical Activity in Kidney Disease: Evidence and Implementation.” Nature Reviews Nephrology 21, no. 12: 846–858. 10.1038/s41581-025-00999-2. 40935901

